# Laparoscopic Partial Splenectomy Assisted by Fluorescence in a 13-Year-Old Girl

**DOI:** 10.1055/s-0040-1716894

**Published:** 2020-10-21

**Authors:** Isabel Bada-Bosch, David Peláez Mata, Manuel de la Torre, Javier Ordóñez, María Dolores Blanco, Juan de Agustin

**Affiliations:** 1 Department of Pediatric Surgery, Hospital General Universitario Gregorio Maranon, Madrid, Spain

**Keywords:** partial splenectomy, laparoscopy, fluorescence, indocyanine green, pediatric

## Abstract

Partial splenectomy allows preserving immune function in benign splenic lesions such as epidermoid cysts. Determining the plane of resection and perfusion of the spleen remnant can be difficult, especially in centrally located lesions. We present a 13-year-old girl with a symptomatic splenic cyst of 6 cm in diameter located next to the splenic hilum. Laparoscopic partial splenectomy was performed through a 10-mm umbilical approach and three accessory 5-mm ports. Intraoperative intravenous injection of indocyanine green (ICG) at 0.2 mg/kg guided the careful dissection of the splenic hilum and checked the spleen perfusion once the upper arterial branch was clamped. The subsequent wash-out of the ICG allowed inspection of the peripheral vascular return of the splenic remnant through polar veins. Surgery was uneventful with minimal blood loss. Follow-up ultrasound scan revealed a well-perfused small splenic remnant with no signs of recurrence.

Laparoscopic partial splenectomy is feasible in benign splenic tumors, especially in those cases of peripheral location. Fluorescence facilitates the safe dissection of the splenic hilum, the visualization of the transection plane of the spleen and the perfusion of the remnant in cases of anatomically and technically complicated partial splenectomies.

## Introduction

Splenic cysts are a rare entity in children, and splenectomy is the first choice. Loss of spleen's immune function is associated with potentially serious complications such as postsplenectomy sepsis. 1 Partial splenectomy allows the removal of the lesion while preserving splenic function. However, it is a difficult technique, especially in large and centrally located cysts. 2 The use of minimally invasive techniques reduces the aggressiveness of the procedure, but there are few cases published of partial splenectomy in children. This is because it is a technically demanding procedure, especially during vascular dissection. Fluorescence facilitates a clearer definition of the anatomy and surgical dissection in technically complicated cases, allowing complex laparoscopic procedures to be addressed with greater safety for the patient.

We present an unusual case of a child with a hilar splenic cyst in which a laparoscopic partial splenectomy guided by indocyanine green (ICG) fluorescence was performed.

## Case Report

**Fig. 1:**
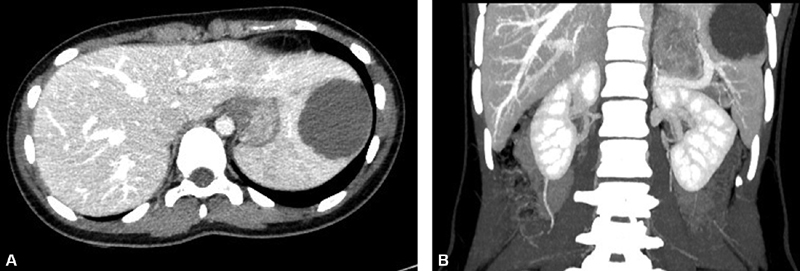
Computed tomography with intravenous contrast. Axial ( **A** ) and coronal ( **B** ) sections in venous phase. A hypodense 48.8 × 55.2-mm lesion with a rounded morphology was observed in the anterior, cranial, and lateral region of the spleen, intimately related to the hilum.

A 13-year-old patient was admitted to our center reporting a 1-year history of diffuse abdominal discomfort and asthenia. She visited the emergency department due to worsening pain and vomiting during the last 24 hours. Abdominal examination and blood tests were normal. An ultrasound scan was performed revealing a 5 × 5.3-cm smooth-edged oval lesion with hypoechoic content, echoes, and thin septa. Magnetic resonance imaging (MRI) and computed tomography (CT) ( Fig. 1 ) revealed a 5.5-cm lesion located in the anterior pole and diaphragmatic face of the spleen, in close relationship to the main superior branches of the splenic artery and vein consistent with an epidermoid cyst. Due to the size of the lesion and the associated symptoms, a surgical approach was favored. The patient received preoperative immunization for meningococcus, haemophilus, and pneumococcus.

The surgical procedure was performed with the patient in supine position with the left side elevated 45 degrees. A 10-mm umbilical Hasson trocar was inserted by open technique and three 5-mm accessory trocars were later placed subxiphoid, epigastric, and on the left flank. Laparoscopic equipment from Stryker Corporation (Michigan, United States) with a led and near-infrared light source (L10 Led Light Source with AIM) and 5 mm 30 degrees optics (1588 AIM) was used. Laparoscopy was performed with a 12 mm Hg CO _2_ pneumoperitoneum with a 4 L/minute flow rate. The surgeon, assistant, and scrub nurse were on the right side of the patient.

**Fig. 2:**
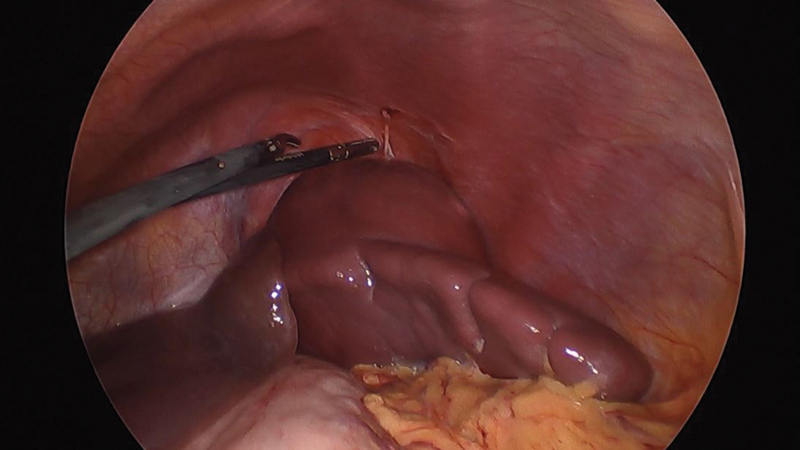
Intraoperative image before beginning dissection. To the left of the image, the left hepatic lobe is seen covering the upper pole of the spleen. The spleen presents a large cyst on the superolateral aspect with diaphragmatic adhesions.

An orthotope spleen with a large peripheral cyst on its lateral side was found. The spleen had some adhesions to the diaphragm and abdominal wall. Small accessory spleens were found on the medial aspect ( Fig. 2 ).

**Fig. 3:**
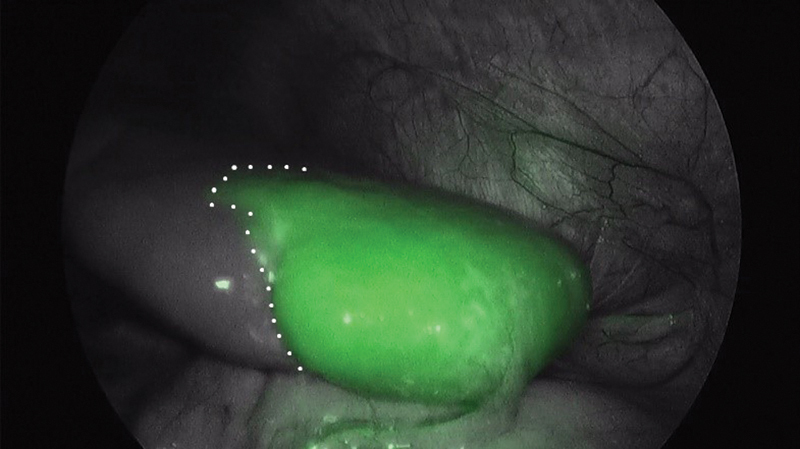
Intraoperative image after arterial clamping. Image under near infrared light. To the right of the dotted line, inferolateral pole of the spleen. It shows fluorescence since arterial vascularization in this portion is preserved. To the left of the dotted line, supero-medial pole. It does not show fluorescence as the superior splenic artery, which supplies arterial irrigation to this portion, has been clamped.

The procedure started with bipolar cauterization and section of the short gastric vessels. Then, dissection of the splenic hilum identifying the splenic artery and vein was performed. After ligature of the superior branch of the splenic artery, the fluorescent dye was administered intravenously. ICG-PULSION 25 mg powder for solution (PULSION Medical Systems, Feldkirchen, Germany) was diluted in 5 mL of bidistilled water to a concentration of 5 mg/mL. From this dilution, a bolus of 0.2 mg/kg was administered. Seventy-two seconds after administration, the contrast was observed in the splenic artery and subsequently fluorescence in the lower-lateral end of the spleen, while the region corresponding to the cyst remained without uptake ( Fig. 3 ).

**Fig. 4:**
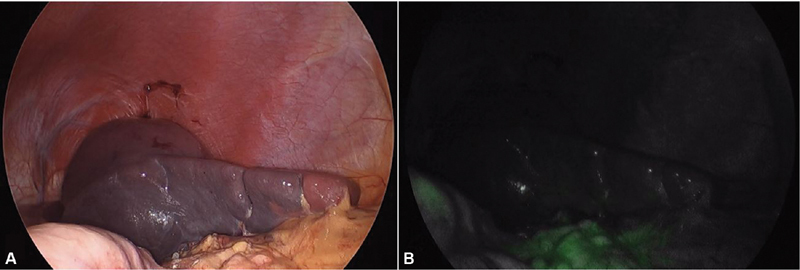
Intraoperative image after venous clamping. ( **A** ) Image under LED light. ( **B** ) Image under near infrared light. No fluorescence is observed in the inferolateral pole of the spleen, reflecting adequate ICG wash-out through a permeable venous system. Some ICG rests can be seen in the omentum (lower part of the image) and stomach (left edge). ICG, Indocyanine green.

**Fig. 5:**
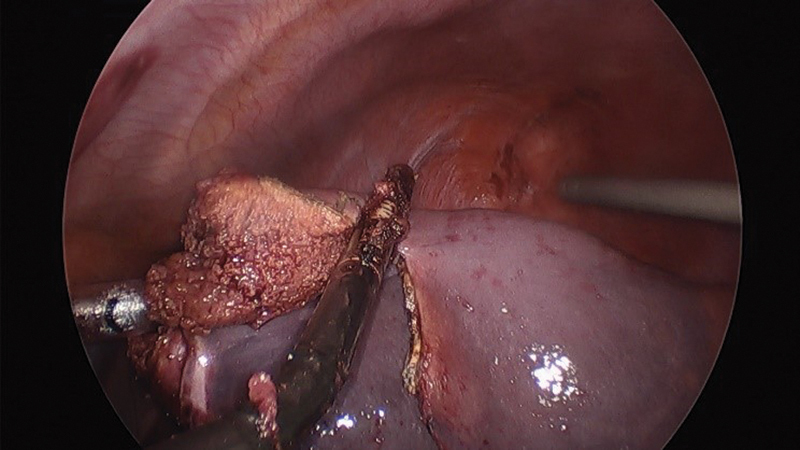
Intraoperative image. Division of the lateral pole from the medial pole using Caiman forceps.

**Fig. 6:**
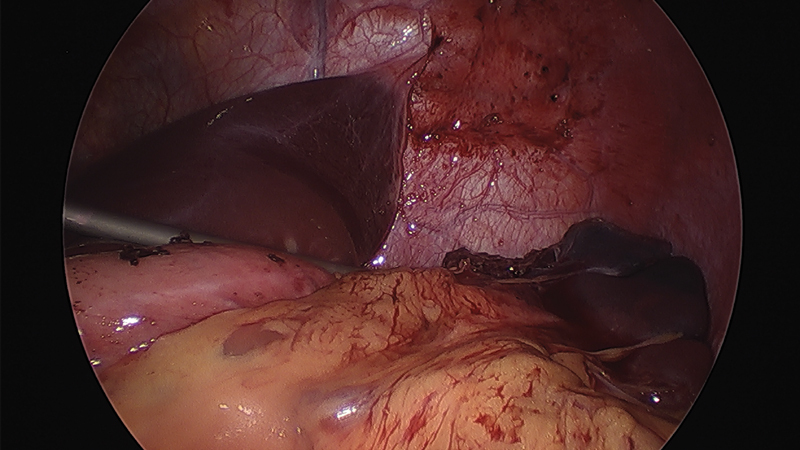
Intraoperative image. Postoperative aspect.

After verifying ICG wash-out through the inferior peripheral polar vessels ( Fig. 4 ), the superior branch of the splenic vein was ligated with Hem-o-lock clip (Auto Endo5, Teleflex, Pennsylvania, United States), ensuring arterial perfusion and venous return of the splenic segment to be preserved. The spleen was divided above the ischemia line using Caiman forceps (B. Braun, Melsungen, Germany) until both territories were separated ( Fig. 5 ). Subsequently, the piece was removed with a laparoscopic bag ( Fig. 6 ).

Pathological anatomy reported a 96 g specimen. Macroscopically, normal splenic parenchyma surrounded a smooth and thin-walled cyst. Microscopic study showed a cyst covered by a nonkeratinized epithelial lining with an immunohistochemical profile corresponding to an epithelial cyst.

The postoperative course was uneventful. The patient did not require blood transfusion and was discharged after 48 hours. Follow-up ultrasound 1 month after surgery revealed a well-perfused small splenic remnant with no signs of recurrence.

## Discussion

Splenic cysts are rare entities. In our setting, up to 90% of primary splenic cysts are epidermoid (nonparasitic), excluding neoplastic cysts. Most primary splenic cysts are diagnosed in children or young adults. Symptoms can range from an incidental finding to life-threatening complications such as rupture, bleeding, or infection. Up to 70% of cysts are symptomatic, 2 with epigastric pain or pain in the left hypochondrium as well as early satiety being the most common complaints. Symptoms are generally correlated with size, 3 with the smallest generally being asymptomatic. 1 2 3 4 5 6

The confirmatory diagnosis is radiological. The initial imaging test is an ultrasound scan with pulse Doppler. CT or MRI allow better anatomical definition and surgical preparation. It is important to carefully delineate the splenic vascularization, both arterial and venous branches. 1 2 3 4 5 6 7

Less aggressive therapeutic alternatives have been described for splenic cysts. Ultrasound monitoring may be useful in simple cysts under 5 cm. Percutaneous aspiration alone or combined with the injection of sclerosing agents, and other surgical procedures such as marsupialization or unroofing have shown a very high recurrence rate, and are also associated with the creation of adhesions that hinder subsequent surgery. Therefore, their use is nowadays discouraged. 1 2 3 4 5 6 7

Splenectomy as treatment of choice is being replaced by partial resection procedures that preserve part of the splenic parenchyma, thus avoiding infectious complications related to the loss of spleen's immune function. Postsplenectomy sepsis is the most serious complication, having a prevalence of 4% of the cases with a mortality of 1.5%. 1 Hence, the preservation of splenic parenchyma is of vital importance, especially in children. Immune function has been shown to be maintained if at least 25% of spleen's parenchyma is preserved. 2 5 However, meningococcus, haemophilus, and pneumococcus vaccination is advised prior to surgery in case total splenectomy is finally performed due to surgical complications. 8

Partial splenectomy is currently considered to be the ideal procedure. Like the vast majority of splenic surgical procedures, the ideal approach is minimally invasive. The dissection of the splenic vessels and their branches continues to be the critical step in this surgery, specially hindered by the broad anatomical variability. For this reason, preoperative angiography is recommended to define the vascular anatomy. 2 In our patient, we performed preoperative angio-CT and used ICG fluorescence to delineate the vascular anatomy in detail during the surgical procedure.

ICG 9 is a dye that, injected intravenously, is distributed through the bloodstream and has complete biliary excretion without recirculation. It is an excellent vascular marker as it quickly reaches well-vascularized tissues and washes out just as quickly if venous return is adequate. It is harmless, cheap, and easy to use. The initial recommended dose is 0.2 mg/kg with the possibility of a second bolus at the same dose. ICG molecules are excited with wavelengths between 750 and 800 nm, emitting fluorescence at 832 nm (near-infrared range) that can be detected by using special cameras built into the laparoscopy system. The use of gray-scale cameras versus others in black and white is recommended, since although both capture ICG fluorescence, the firsts improve the visualization of the surgical field during the procedure and facilitate vascular dissection. 10

Limitations to the use of ICG are scarce. In patients with abnormal liver function the uptake and especially, the elimination may be altered. It is contraindicated in those allergic to iodinated contrasts or with thyroid disorders. 9 11 12 13 14

The use of fluorescence in surgery is emerging, but experience in splenic pathology is very limited. Kawasaki et al 15 used ICG during distal pancreatectomies with splenic preservation. Aggarwal et al 16 described a cystectomy using ICG to contrast the splenic parenchyma against the cyst, which being avascular did not uptake contrast. Only Mizuno et al 17 present a case of partial splenectomy following a procedure similar to ours in a 50-year-old man. To this date, we have not found any published articles on the use of ICG in procedures on splenic parenchyma in children.

We consider that the use of fluorescence provides advantages over conventional laparoscopic partial splenectomy. It facilitates vascular dissection by defining the anatomy in real time, it demonstrates adequate arterial perfusion of the remaining tissue, and it increases the safety of a technically complicated procedure, without increasing the surgical time and without notable drawbacks.

## Conclusion

When surgical resection of epidermoid cysts is indicated, we believe that the ideal technique is laparoscopic partial splenectomy, allowing the removal of the lesion while preserving the splenic parenchyma. Dissection of the splenic hilum is the key step during the procedure. The use of ICG-mediated fluorescence facilitates dissection and allows the vascularization of the splenic remnant to be checked, which increases the safety of the procedure.

To the best of our knowledge and by going through the previous publications in PubMed, this is the first article regarding the use of fluorescence during a laparoscopic partial splenectomy in a pediatric patient.

## Notes

New Insights and the Importance for the Pediatric Surgeon Laparoscopic partial splenectomy is a technically challenging procedure for the pediatric surgeon. The use of indocyanine green-mediated fluorescence facilitates dissection and allows the vascularization of the splenic remnant to be checked, which increases the safety of the procedure.
